# The elimination of stressed induced light leakage for in-plane-switching LCD

**DOI:** 10.1038/s41598-022-07182-8

**Published:** 2022-02-28

**Authors:** Feifei Wang, Hongming Zhan, Lintao Ji, Tao Yang, Qingyong Meng, Yajun Li, Bowen Li, Yuansheng Zang, Junsheng Chen, Yinhu Huang, Kaixuan Wang, Lifeng Lin, Xibin Shao

**Affiliations:** BOE Technology Group Co., Ltd., No.12 Xihuanzhong RD, BDA, Beijing, 100176 People’s Republic of China

**Keywords:** Materials science, Optics and photonics

## Abstract

As the mainstream display mode of LCD, IPS is overwhelmingly used in many fields of flat displays. However, due to the stress sensitivity of glass, the stressed light leakage is a bottleneck for achieving perfect dark state performance. The conventional scheme of using a compensation polarizer outside the cell has no effect on this light leakage. Although many studies have been conducted to overcome this limitation, the proposed methods have limited effects. Our research team has proposed a novel light leakage compensation mechanism by introducing a positive A plate that is sandwiched between the glass and the LC layer, therefore the light leakage which is caused by the combined effect of the phase retardations from the stressed glasses and the LC layer can be eliminated. In addition to theoretically analyzing the compensation principles of the novel light leakage compensation mechanism, we also use the developed positive A material to prepare light leakage compensation demos. And then the electric-optical characteristics and light leakage compensation effects of the demos are evaluated. While maintaining excellent optical and electrical characteristics, this technology effectively solves the problem of stressed light leakage of glass-based IPS, improves the dark-state image quality, and breaks the application of IPS in products such as curve products.

## Introduction

After decades of development, IPS (In-Plane Switching) LCD (Liquid Crystal Display) occupies a dominant position in the display field. Due to its excellent display performance, IPS is widely used in all sizes of display products, such as mobile phones, tablet computers, notebook computers, monitors, and TVs^1–9^. With the continuous expansion of the display technology application field, especially the application of curve products, ultra-large size products, automotive, medical, and anomalous screens, the requirements for display technology are getting higher and higher. Dark-state light leakage (LL) is a problem that has always plagued glass-based IPS. Solving the LL can effectively improve the customer experience, greatly enhance competitiveness and break through the limitations of IPS applications in various fields. So it is of great significance^10–14^.

Based on previous researches on dark state LL of IPS ^15–21^, although there are many factors that cause LL in the dark state (peripheral stress, PS design, LC arrangement, etc.), the most important and fundamental reason for glass-based IPS is the stress-birefringence of CF/TFT (Color Filter/Thin Film Transistor) glass. he stress birefringence of the glass, which can be expressed as^16–18^:

1 $$\updelta =\mathrm{SOC}\times\upsigma \times \mathrm{T}$$

where δ is the stressed retardation of glass, SOC is the stressed optical coefficient, σ is the stress, T is the thickness of glass. So the retardation value is proportional to σ and T.

The Tr of horizontal electric field mode can be expressed as follows^22^:

2 $$\mathrm{Tr}=1/2\times {\mathrm{sin}}^{2}2\mathrm{\varphi }1 \times {\mathrm{sin}}^{2}\left(\uppi \times\updelta 1/\uplambda \right)$$

where φ1 is the angle between the direction of the electric field and the optical axis of LC, δ1 is the retardation of LC, λ is the wavelength of light.

The LL is proportional to the backlight luminance and the transmittance (Tr) of the panel. When evaluating the LL caused by stress, the backlight luminance and the φ are fixed, so the LL is proportional to Tr. At this time, the δ1 in the Tr formula becomes the retardation related to stressed glass and LC, it is proportional to the retardation of glass (δ). Therefore, LL can be derived as:

3 $$\mathrm{LL }\propto {\mathrm{sin}}^{2}\left(\uppi \times\updelta /\uplambda \right)$$

Due to the stress birefringence of the glass is very small, the π × δ/λ is much smaller than π/2, so the LL can be expressed as follows:

4 $${\mathrm{LL }\propto {\updelta }^{2}=(\mathrm{SOC}\times\upsigma \times \mathrm{T})}^{2}$$

It can be known that the LL is proportional to the square of δ. Consider the formula (1), LL is also proportional to the square of σ and the square of T as shown in formula (4).

Due to the manufacturing process of CF/TFT glass and panel, and even the using process of IPS panel, it is difficult to completely avoid the stress birefringence of glass. Although some studies have proposed solutions, such as slimming of glass thickness^17,18^, reducing of retardation of LC, increasing of photo spacer support density^19^, using a local dimming display system consisting of segmented backlight and a LCD panel^23,24^. These methods have limited functions and cannot solve the dark state LL completely.

We have been committed to the research of completely resolving the inherent LL of glass-based LCD^25^. Considering IPS is the mainstream technology in LCD, subsequent simulation and research in this article are based on IPS mode. And our reach is focused on the stress LL caused by the mechanical deformation stress. Generally, the mechanical stress of the panel produces from the assembly process for cell and module respectively. For example, large-size products, curved products, etc., the panel will experience mechanical deformation stress and light leakage, and deformation can be simply described as bending. Referring to previous reports^17,21^, the CF glass is a concave surface and it experiences compression, the TFT glass is a convex surface and it experiences tension. Under pure bending, the tensile and the compressive stresses are equal in magnitude but opposite in direction. The bending stress can be calculated^17,26^ using:

5 $$\sigma =\frac{\mathrm{Et}}{2r}$$

where σ is the stress from bending, E is Young’s modulus of glass (73,000 MPa for LCD display used glass), t is the thickness of the glass sheet and r is the radius to which the sheet is bent. In an ideal case, as the light passes through the bent glass, the in-plane retardation can be calculated by the stress-optic law shown in formula (1).

In this article, referring to the fundamental reason for light leakage, we have proposed a novel light leakage compensation mechanism, and a new LCD structure with an in-cell phase retarder as a solution. The basic idea of phase compensation is to introduce a positive A (+A) plate to compensate for the retardation of LC, make the stress birefringence of CF and TFT glass offset each other, and effectively eliminate light leakage. We have explained the compensation mechanisms, analyzed the electric-optical characteristics, and studied the effects of LL compensation. It is a very important point to note that, different from studies of compensation layers on improving the viewing angle^27,28^ and response time^29^, the compensation layer for glass stress leakage must be placed between the upper and lower glass, inside the cell. The conventional scheme of using a compensation polarizer outside the cell has no effect on this stressed light leakage. This is because the conventional viewing angle compensation mainly considers the color shift problem caused by the difference of LC phase retardation at large viewing angles. At this time, the glass is regarded as the isotropic layer. There is no difference whether the compensation position is outside or inside the glass, just between the PVA layer of up and down POL is ok. In the stress light leakage problem, the glass becomes an optically anisotropic layer under stress. The purpose of the light leakage compensation structure mentioned in this article is to compensate for the light leakage problem caused by the combined effect of the glass phase retardation and the LC phase retardation. At this time, to ensure the effectiveness of light leakage compensation, the compensation layer must be placed between the up and down glass. At present, the compensation POL is easier to realize and is widely used. The commonly used  +A plate, fabricated using uniaxially stretched polymer films, such as polyvinyl alcohol (PVA), cyclic olefin polymer (COP), cyclic olefin copolymer (COC), or other suitably oriented organic birefringence materials, usually used in combination with a polarizer to achieve viewing angle compensations. Limited by process conditions, these kinds of  +A materials cannot be used to prepare an in-cell compensation layer. Thus, in addition to theoretical analysis, another important work is that we have cooperated with JNC CORPORATION to develop the  +A materials. This material can be made as a compensation layer both in or out the glass. And then we conducted an in-depth study on the compensation effect of the new LCD demos. This new LCD structure can effectively solve the bottleneck problem of dark state LL of glass-based IPS, and keep the original photo-electric characteristics of the panel unchanged. In addition, the research in this manuscript breaks the limitations of existing materials and processes, it has far-reaching significance for IPS to enhance its competitiveness.

## Results

**Figure 1:**
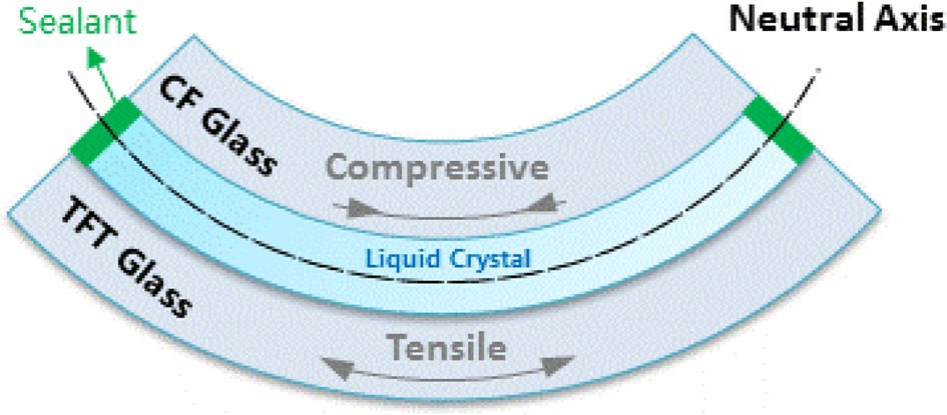
Diagram of panel bended.

As mentioned above, our research focuses on stressed LL caused by mechanical deformation stress. When the panel is under force, due to the fixing effect of the sealant, the panel as a whole, it experiences tension on the TFT glass and compression on the CF glass as bending ^17,21,26^. As shown in Fig. 1, under pure bending, these tensile and compressive stresses are equal in magnitude but opposite in direction. At the neutral axis where the transition between tensile and compressive zones occurs, the stress is zero.

In order to analyze the stress leakage mechanism of IPS and the leakage compensation mechanism proposed in this paper, we calculated the stress value and the stressed retardation of the glass and carried out the relevant simulation and analysis. According to formula (5) and formula (1), when the glass thickness is 0.3t and the radius of curvature is 1200 mm, the σ is 9.13Mpa, the retardation δ produced by the stress birefringence of the CF and TFT glass is 9.24 nm, and the direction is perpendicular to each other.

### The stressed light leakage mechanism

**Figure 2:**
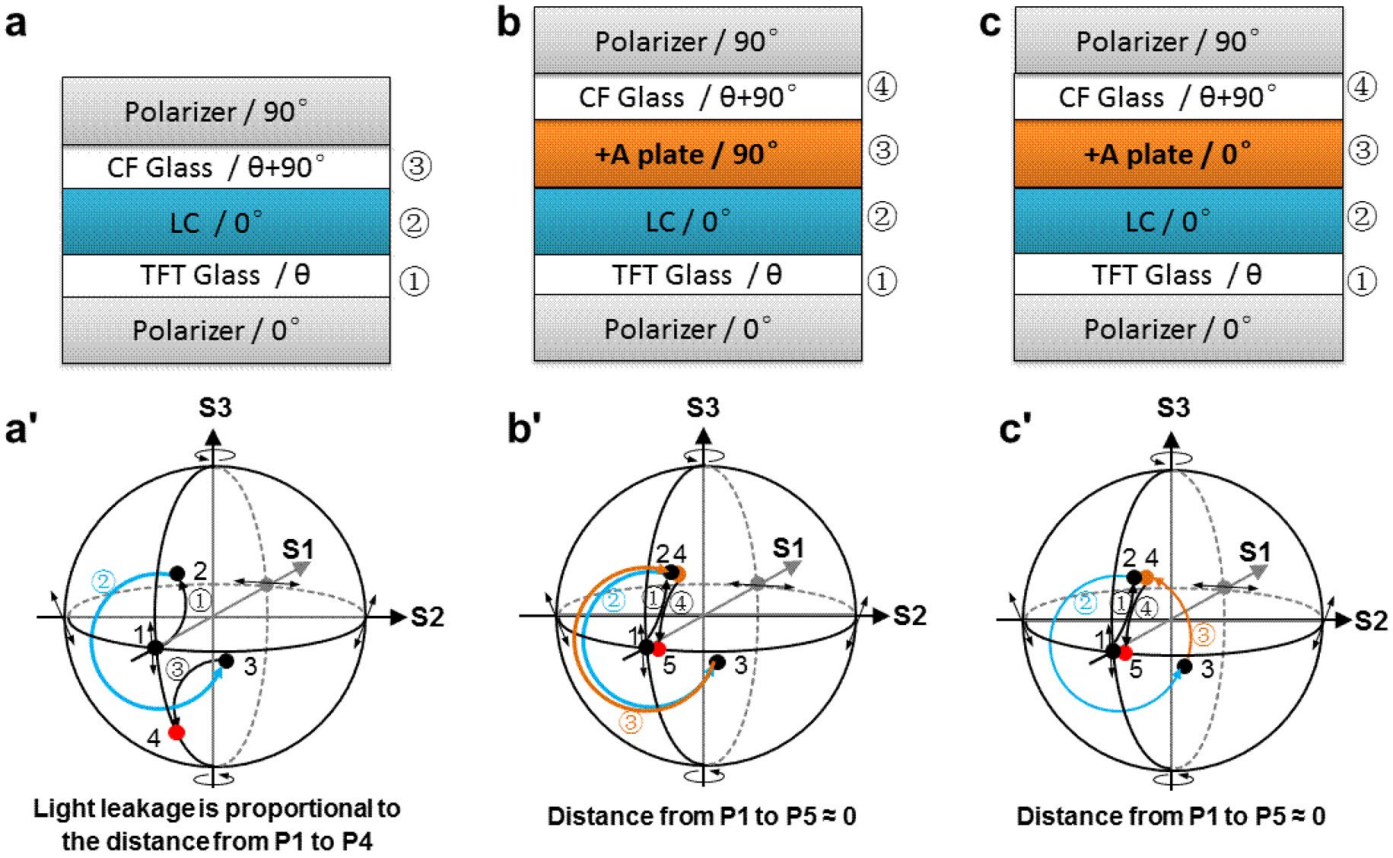
The compensation principle of the new IPS. (**a**) The structure of normal IPS. (**a**') The stressed LL principle of normal IPS. (**b**) The structure of compensation mode 1. (**b**') The compensation mechanism of compensation mode 1. (**c**) The structure of compensation mode 2. (**c**') The compensation mechanism of compensation mode 2.

And as shown in Fig. 2a, the angle between the optical axis of TFT glass and LC is θ, and the angle between the optical axis of CF glass and LC is θ + 90°. The residual stress of the glass or the uneven stress caused by the frame during the IPS manufacturing process will cause the glass to produce retardation. And the brightness of the LL is proportional to the square of phase retardation (δ). After the stressed retardation of glass is got, the LL can be confirmed by simulation software.

The stressed LL mechanism of normal IPS is illustrated by using the Poincaré sphere^28^. The Poincaré sphere based on Stokes is usually used to characterize analyze the polarization state of light. As shown in Fig. 2a', PI (Point 1), P2 (Point 2), P3 (Point 3), and P4 (Point 4) respectively represent the polarization state of light after passing through the polarizer, the TFT glass, the LC layer, and the CF glass. Due to the effect of phase retardation of LC, a certain level of LL occurs. When the angle between the optical axis of LC and the glass with stress birefringence is 0° or 90°, the phase retardation of the LC is invalid, and there is no light leakage. But when the angle between the optical axis of LC and the optical axis of the glass with stress birefringence is not 0° or 90°, the vertically incident light becomes linearly polarized light after passing through the TFT polarizer, due to the effect of the LC phase retardation, the polarization state after passing through the TFT glass, the LC layer, and the CF glass is changed. When passing through the CF polarizer, it cannot be completely absorbed and LL occurs. As shown in Fig. 2a', the distance from P1 (Point 1) to P4 (Point 4) is proportional to LL brightness.

### The compensation mode 1

Due to the combined effect of the phase retardations from the stressed glass and the LC layer, the existing IPS structure cannot eliminate the influence of glass stress. The key to solving this problem is to ensure that the light is located at P2 (as shown in Fig. 2a') before the light reaches the CF glass. We have proposed two compensation structures based on IPS mode. These compensation structures with an additional optical layer that can be matched with LC, and effectively eliminate light leakage. Although both schemes introduce a  +A plate and effectively eliminate LL at a dark state, they have different structures and mechanisms.

As shown in Fig. 2b, the first new LCD structure called compensation mode 1, introduces the  +A plate, which is sandwiched between the glass and LC. More specifically, the optical axis of the  +A plate is perpendicular to the initial optical axis of LC, and the phase retardation of the  +A plate is 350 nm, which is equal to that of the LC.Fig. 2b' illustrates the compensation principle of compensation mode 1. When receives external stress, the light from the backlight unit traverses the TFT polarizer, the effective optical axis position on the Poincaré sphere is P1, when the light (P1) successively passes through the stressed TFT glass, its polarization state is rotated from P1 to P2. And when the light (P2) passes through LC, its polarization state is rotated from P2 to P3. Then, the light (P3) successively passes through the  +A plate and the stressed CF glass, whose effective optical axis positions on the Poincaré sphere are P4 and P5, respectively. The intermediate polarization state (P2) in general, is an elliptical polarization state. Due to the role of the  +A plate, the polarization state (P5) on the Poincaré sphere is very near to the polarization state (P1), so the light almost can be absorbed by the CF polarizer, and the elimination of LL is achieved.

### The compensation mode 2

The second new LCD structure called compensation mode 2, also introduces a  +A plate, but the optical axis of the  +A plate is parallel to the initial optical axis of LC, the sum of the phase retardation of  +A and LC is an integer multiple of a specific wavelength. Considering that the human eye has the strongest sensitivity to green light, the retardation value of  +A is designed to be 200 nm, and the sum of phase retardation of  +A and LC is 550 nm, that is the specific wavelength is 550 nm.

The compensation structure of mode 2 is shown in Fig. 2c. The main difference between mode 1 and mode 2 is reflected in the role of the  +A plate. For mode 1,  +A plate realizes that the light follows the same path as LC, and returns back in the opposite direction to the same polarization state as the light before the incident LC. For mode 2, the  +A plate realizes that the light continues along the same direction as the LC and moves forward with a certain optical path to the same polarization state as the light before the incident LC. So for mode 2, as shown in Fig. 2c', due to the role of  +A plate, the polarization state (P5) on the Poincaré sphere is very near to the polarization state (P1) of the light passed through the TFT polarizer, so the light almost can be absorbed by CF polarizer and the LL compensation is realized.

### V-T curve

The electro-optical characteristics of IPS, such as the dark state brightness and V-T curve, are usually studied by the TechWiz (Korea Sanayi System Company) software which is based on Extended Jones.

**Figure 3:**
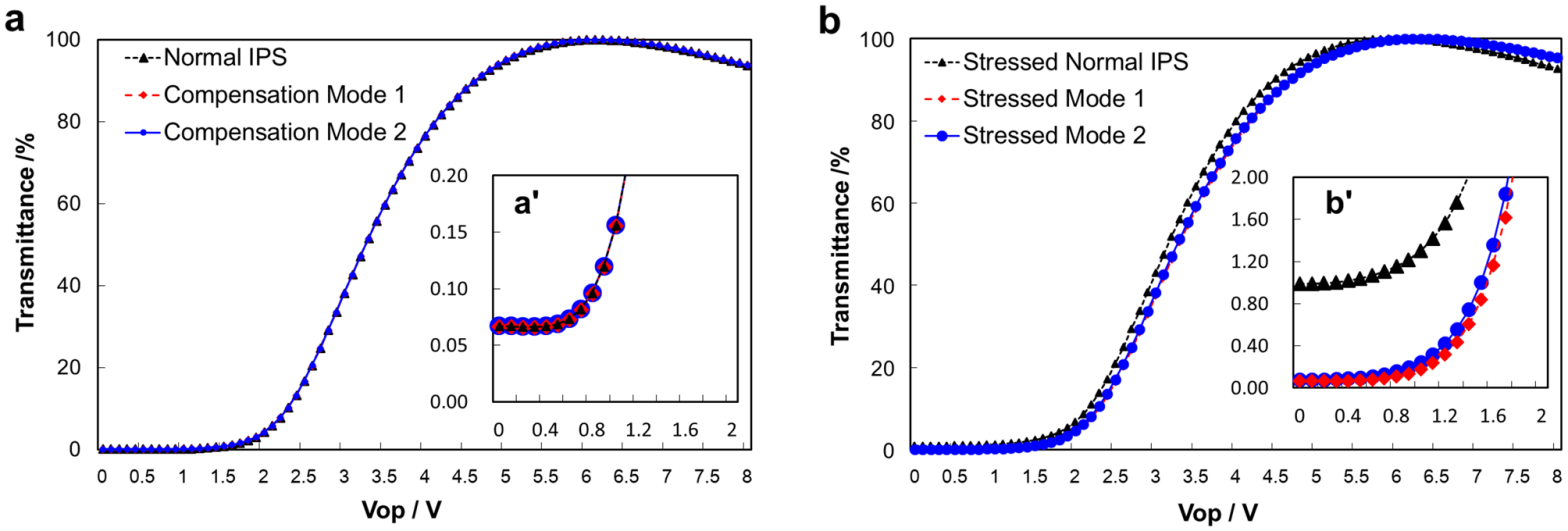
(**a**) The V-T curves of normal IPS, compensation mode 1 and mode 2. (**a**') The partial enlarged V-T curves of (**a**). (**b**) The V-T curves of stressed normal IPS, stressed compensation mode 1 and mode 2. (**b**') The partial enlarged V-T curves of (**b**).

The simulated V-T curve is shown in Fig. 3a. The V-T curve of normal IPS and compensation mode 1 and mode 2 are basically the same. As can be seen from the enlarged picture Fig. 3a’, the dark state brightness without the additional stress of normal IPS, compensation mode 1 and mode 2, is basically the same. But when the additional stress is applied, the difference between normal IPS and compensation modes appears. As shown in Fig. 3b, after being subjected to external stress, the V-T curves of normal IPS, compensation mode 1, and mode 2 have a slight shift. The main difference is reflected in the dark state brightness. It can be seen from the enlarged picture Fig. 3b', the transmittance of the dark state rises from 0.067% to 0.987%, which means the LL occurs in normal IPS. And the transmittances of the compensation modes at the dark state remain almost unchanged (0.067%). So we can conclude that the compensation mode can effectively resist the dark state LL caused by external stress.

When the thickness of the glass is not equal, such as the thickness of the CF glass is 0.3t and the thickness of the TFT glass is 0.4t, the panel as a whole still has the same compressive and tensile stresses on the neutral axis, but the neutral axis is not in the middle of the CF and TFT glass. When the neutral layer is located in the TFT glass, the CF glass has compressive stress. But the TFT glass has tensile and compressive stress, as the stress birefringence δ generated by each has different directions, the δ_compressive_ of TFT can be offset by the δ_tensile_ of TFT, and finally, the δ_tensile_ of TFT will be equal to the δ_compressive_ of CF glass. At this time, the situation is the same as when the thickness of TFT and CF glass is equal. So when the stresses of the CF and TFT glass are not completely equal, the proposed compensation mode can still effectively reduce light leakage.

### Viewing angle

**Figure 4:**
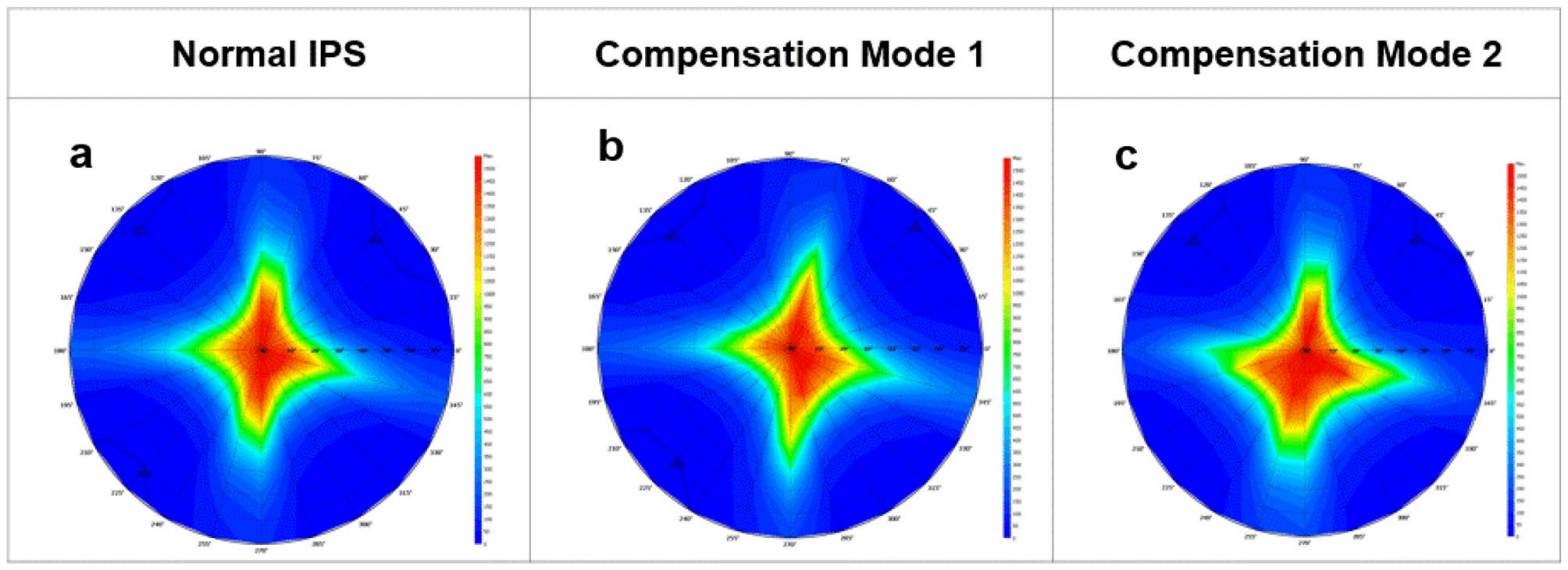
The CR of (**a**) normal IPS, (**b**) the compensation mode 1, and (**c**) the compensation mode 2.

By comparing the viewing angle results in Fig. 4, the viewing angles of normal IPS and compensation mode 1 and mode 2 in the horizontal and vertical directions are almost equivalent, but for other viewing angles of compensation mode1 and mode 2 are different from the reference. This is mainly due to the effect of the  +A layer. For mode 1, since the optical axis of LC and  +A are perpendicular to each other, it is slightly worse than normal IPS at large viewing angles. For mode 2, the  +A and LC optical axes are parallel, the difference in viewing angle is aggravated, but this has little effect, mode 1and mode 2 can still meet the viewing angle specification of 89°/89°/89°/89°.

## Discussion

### Materials design

Normally, +A film is an anisotropic birefringence film with only one optical axis. The refractive index ellipsoid of the uniaxial  +A film: n_x_ > n_y_ = n_z_. The shadow plane represents the film’s surface plane, which is parallel to the X–Y plane. From the viewpoint of optical axis orientation, and the  +A film’s optical axis is parallel to the film surface. +A plate is a commonly used uniaxial birefringence film for phase compensation. It can be fabricated by the use of uniaxially stretched polymer films, such as polyvinyl alcohol (PVA), cyclic olefin polymer (COP), cyclic olefin copolymer (COC), or other suitably oriented organic birefringence materials. And +A plate is usually used in combination with a polarizer to achieve viewing angle compensation.

According to the LL principle of the glass-based IPS and the compensation principle of the compensation mode, the  +A film is required to be placed between the two glass substrates to compensate for the LC phase retardation. Integrating the display specification requirements of IPS products, a series of requirements are put forward for  +A materials. Firstly, from the aspect of process manufacturing, it is required that the  +A film should be prepared by IPS’s coating equipment, and the thickness of the  +A film should be as thin as possible. Secondly, in terms of characteristics, the  +A film is required to have excellent optical properties, such as transmittance and CR, and have good process stability and reliability. Therefore, the  +A material is required to be a polymer material with LC characteristics. Although LC polymer materials have been extensively studied due to their good optical properties^30,31^, there are currently no mature materials that can be directly applied into IPS cells through the IPS manufacturing process.

In order to meet mentioned characteristics above, we have developed LC polymer materials in cooperation with JNC CORPORATION. The developed LC polymer materials contain several LC polymerization monomers, free radical photoinitiators, solvents, and a small number of additives (surfactant, free radical scavengers). The solvent used is CHN (Cyclohexanone). Among the above components, the most important is the LC polymerizable monomer.

**Figure 5:**
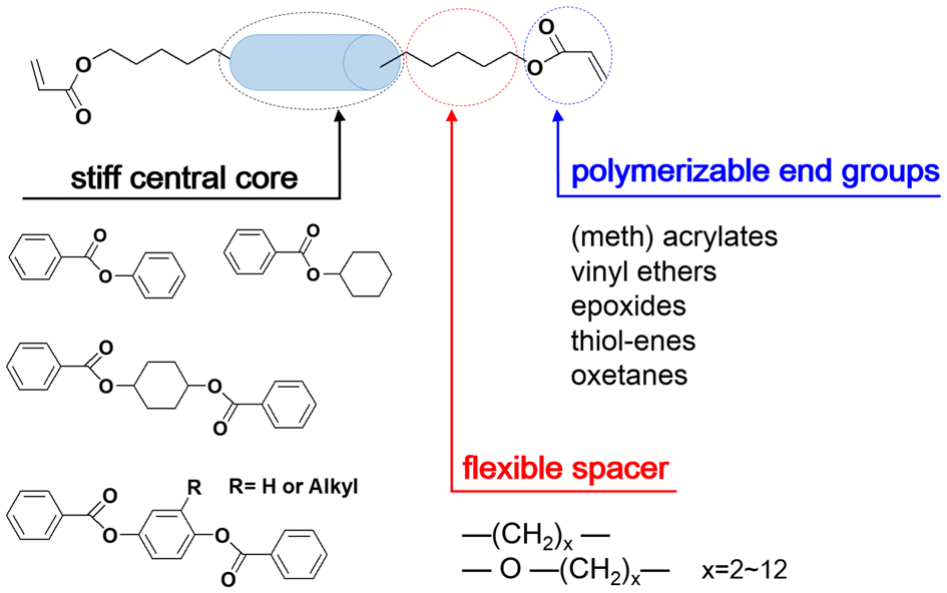
Schematic illustration of the formation of the LC monomer.

As shown in Fig. 5, it is a schematic diagram of the structure of a LC polymerized monomer. In addition to the stiff central core and the flexible spacer required by conventional LC materials, polymerizable end groups are added. Among the end groups, acrylic end groups are widely used due to their higher degree of polymerization with a small amount of initiator. The physical properties of the LC are achieved by adjusting these structural units and their combinations^32^. After verifications and optimizations,  +A materials have excellent processing and optical characteristics. The main characteristics of the  +A material are listed below: birefringence is 0.13 ~ 0.18, viscosity is 2 ~ 5mpa·s, and clearing point (T_ni_) is 100 ~ 140 degrees centigrade, which can meet the optical characteristics and reliability requirements of IPS.

Furthermore,  +A film is mainly prepared^33^ by coating, pre-bake, UV cure, and post-bake processes on the TFT or CF substrate. The preparation processes and conditions of the  +A film layer meet the technological requirements of IPS existing equipment. Therefore, we prepare  +A samples and conduct related research.

**Figure 6:**
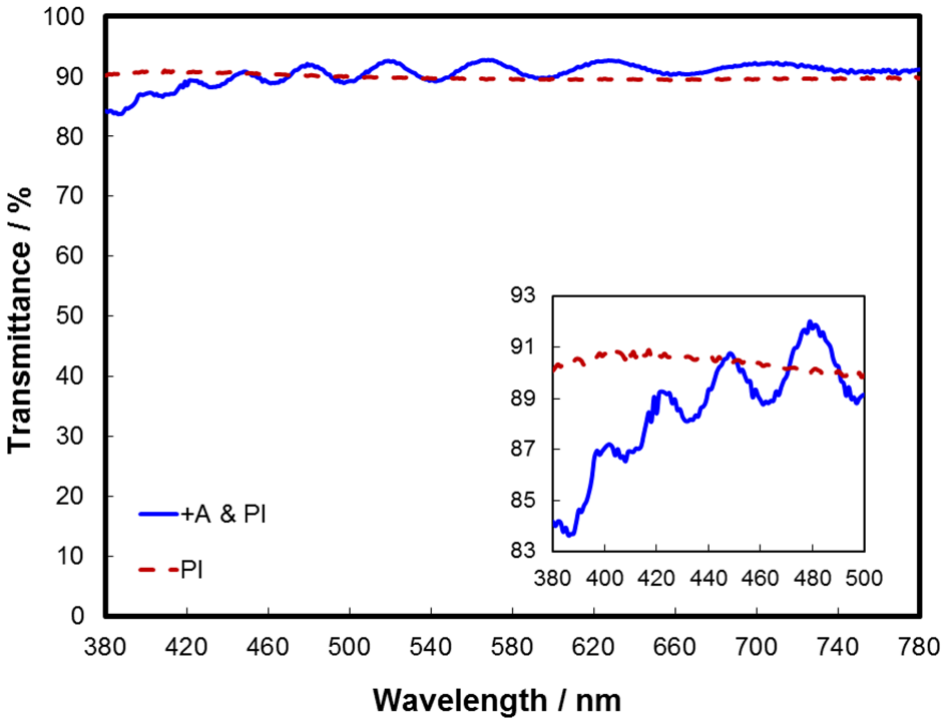
The transmittance curve of the substrate before and after coating the  +A plate.

The transmittance of  +A is tested as shown in Fig. 6, the transmittances of the polyimide (PI) substrate before and after coating  +A are basically unchanged at 450 ~ 780 nm, while the transmittances of the substrate with  +A at 380 ~ 450 nm are slightly lower than that of PI substrate.

### Electric-optical characteristics

In order to study more deeply, we prepared 3.54inches (75.78 mm × 49.02 mm, resolution 480 × 320) demos and conducted related investigations on electric-optical characteristics, including transmittance, Operating Voltage (Vop), RT (response time), CR (contrast ratio), and the compensation effect.

**Figure 7:**
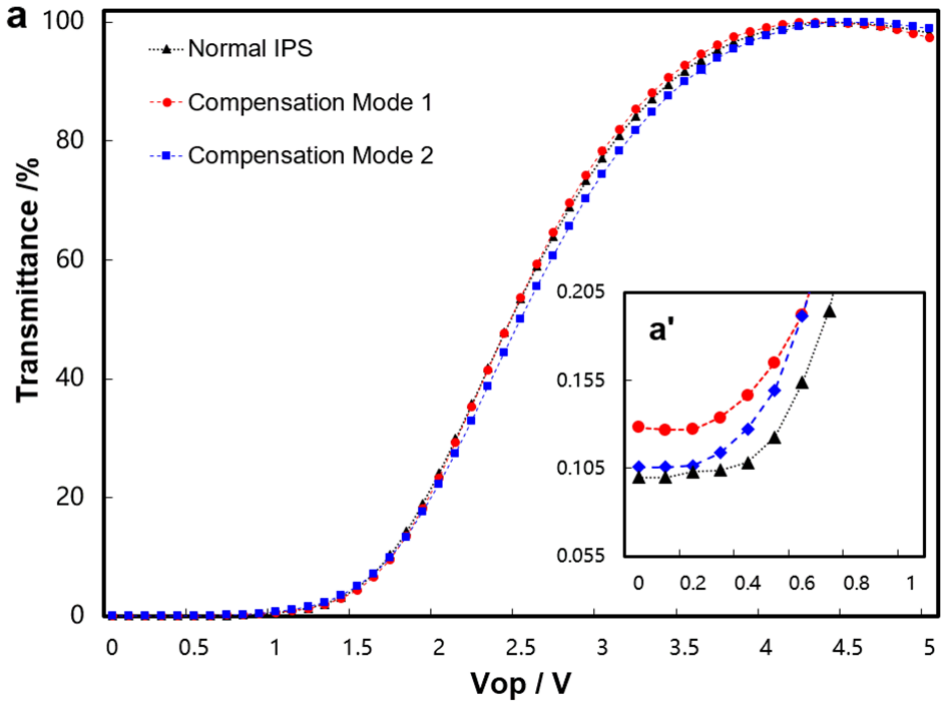
(**a**) The V-T curves of normal IPS, compensation mode 1 and mode 2. (**a**') The partial enlarged V-T curve of (**a**).

The electric-optical characteristics of the panel are measured by DMS-1250 (Autronic Melchers Company). Figure 7 shows the V-T curves of 3.54inches demos. Because the light dispersion effect of LC and other materials was not considered in the simulation, the simulated value and the actual value cannot be completely consistent, but the trends and conclusions of the two are consistent. As can be seen from Fig. 7a, the V-T curves of normal IPS, compensation mode 1, and mode 2 basically coincides. Furthermore, it can be seen from the enlarged picture Fig. 7a', the dark state brightness of the sample is basically the same when no external force is applied, and the dark state brightness of compensation mode 1 is somewhat higher. The transmittance of normal IPS, compensation mode 1, and mode 2 are 0.099%, 0.128%, and 0.106% respectively. The transmittance curves are obtained from the brightness of the gray scales. The L0 brightness of normal IPS, compensation mode 1, and mode 2 are 0.5769nit, 0.6774nit, and 0.5526nit, respectively. Because the brightness test accuracy of the equipment is ± 0.01, the device error can be eliminated. It can be seen that the difference in brightness and transmittance is mainly caused by sample differences. The L0 brightness of ordinary IPS and mode 2 is basically the same, while the L0 brightness of mode 1 is higher.

The other test results of electric-optical characteristics are listed in Table 1, the transmittance of normal IPS, compensation mode 1, and mode 2 are 5.76%, 5.32%, and 5.39%, respectively. And the lower transmittances of compensation mode 1 and mode 2 are mainly affected by the low transmittance of  +A at 380 ~ 450 nm. The Vop of normal IPS, compensation mode 1 and mode 2 are all 4.4 V, respectively. And the RT of normal IPS, compensation mode 1 and mode 2 are 16.67 ms, 16.38 ms and 16.64 ms, respectively.

**Table 1:** The electric-optical characteristics.

Item	Normal IPS	Compensation mode 1	Compensation mode 2
Transmittance	5.76%	5.32%	5.39%
Vop	4.4 V	4.4 V	4.4 V
RT	16.6 ms	16.38 ms	16.64 ms
CR	1002	789	993

The CR of compensation mode 1 is 789, lower than that of normal IPS (1002). The CR of compensation mode 2 is 993, which is basically the same as that of normal IPS. So for compensation mode 2, besides improving the image quality of the dark state, it maintains the original technical advantages of normal IPS in terms of electric-optical characteristics.

For compensation mode 1, the dark state brightness is higher than that of the other two demos as shown in Fig. 3, so it has a lower CR. General, the light scattering of LC is an important factor to increase dark-state brightness and reduce panel CR^33–35^. And the intensity of scattered light of LC layer is proportional to the thickness of LC layer^12,36,37^.

**Figure 8:**
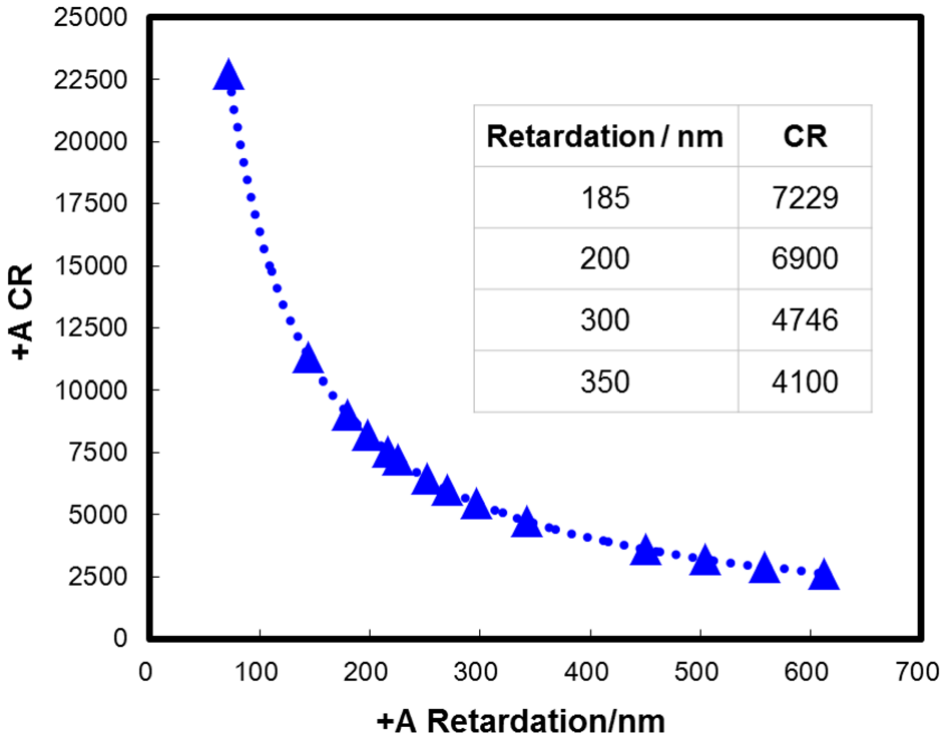
The CR of  +A at different retardation.

Since the  +A plate is also horizontally arranged LC, its light scattered characteristics are similar to those of LC materials. The intensity of scattered light of the  +A layer is proportional to the thickness d of the  +A layer. As the retardation of  +A is Δn*d, the intensity of scattered light of  +A is also proportional to retardation. The CR of  +A films are tested by CF Contrast Tester from Denkei. Figure 8 shows the CR of the  +A films. The CR of  +A decreases as the retardation increases. This is mainly because the retardation is proportional to d, the increase of d will increase the intensity of scattered light and decrease the CR of  +A film.

**Figure 9:**
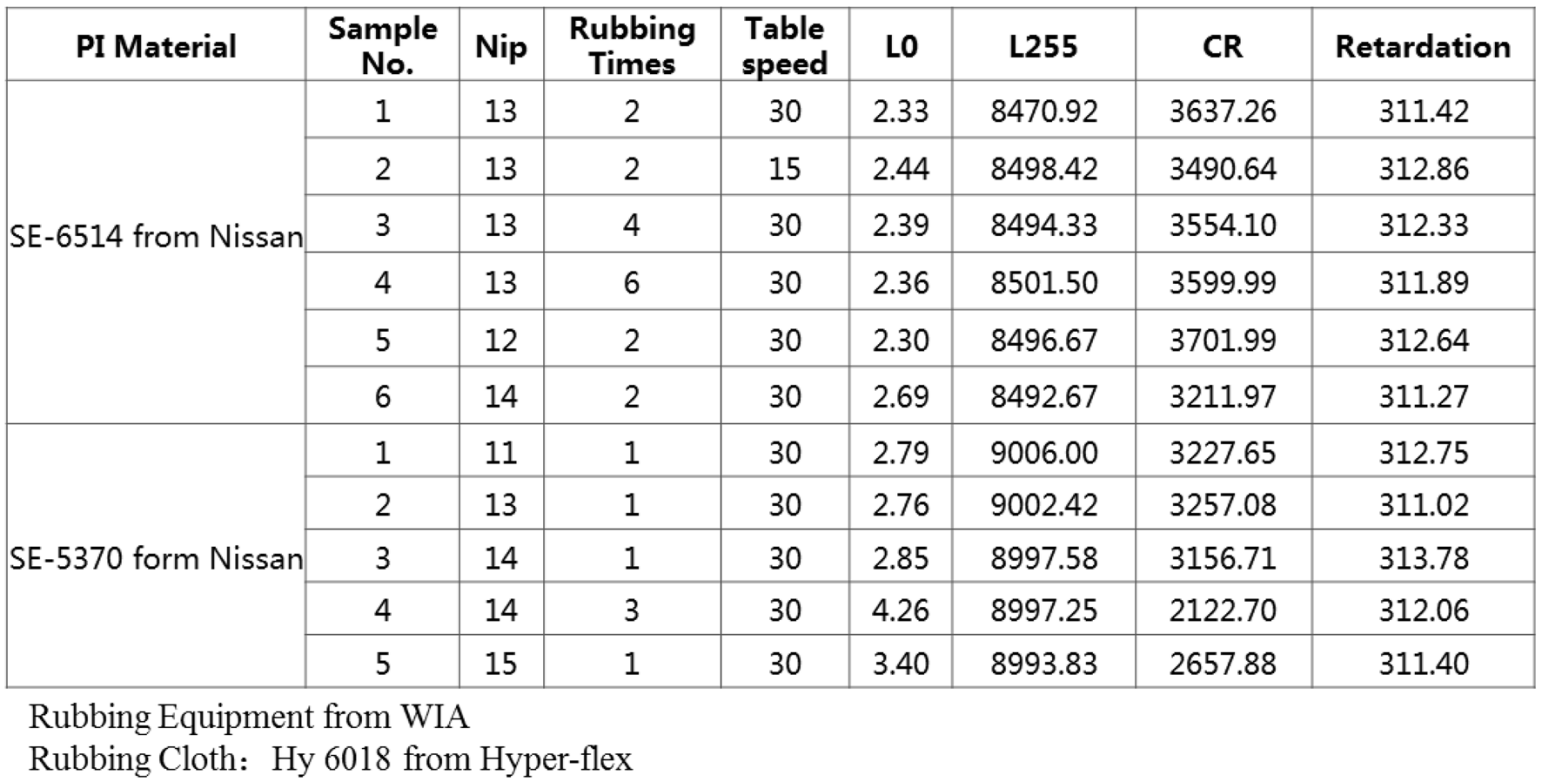
The CR of  +A at different alignment condition.

Since the LC polymer needs to be aligned to form a  +A plate, the alignment quality will affect the optical properties of  +A, especially the CR of  +A film. So we compare the effects of different alignment conditions on CR. It can be seen from Fig. 9 that alignment optimization can be used as a method to improve  +A CR.

Table 2 are the CR of  +A and panels. The retardations of compensation mode 1 and mode 2 are 350 nm and 200 nm, respectively. Due to the effect of light scattering, the CR of mode 1 (4100) is lower than that of mode 2 (6900). For mode 1, the low CR of  +A is the bottleneck CR among the various optical layers of the panel and further reduces the CR_panel_ to 789. So it is necessary to increase the CR_+A_ for mode 1. For mode 2,  +A has a higher CR of 6900 which is almost equivalent to the color filter CR in IPS, the CR_panel_ remains the same as normal IPS. Based on the existing compensation materials, compensation mode 2 is recommended.

**Table 2:** The characteristics of compensation layers.

Item	+A		Panel
	Retardation	CR	CR
Normal IPS	–	–	1000
Mode 1	350 nm	4100	789
Mode 2	200 nm	6900	993

### The light leakage compensation

#### The planar demos

Therefore, in order to further study the dark state LL compensation effect of compensation technology, we have prepared the 13.3inches (293.76 mm × 165.24 mm, resolution 2160 × 1080) demos of compensation mode 2, and carried out related researches on the effects and influencing factors of light leakage. First, apply additional stress to 13.3inches demos using the pressure gauge, and then use optical equipment CA310 to test the brightness of the sample before and after the extra force. At last, we compared and analyzed the LL results of normal IPS and compensation, and conducted a more in-depth study on the factors that affect the LL compensation.

**Figure 10:**
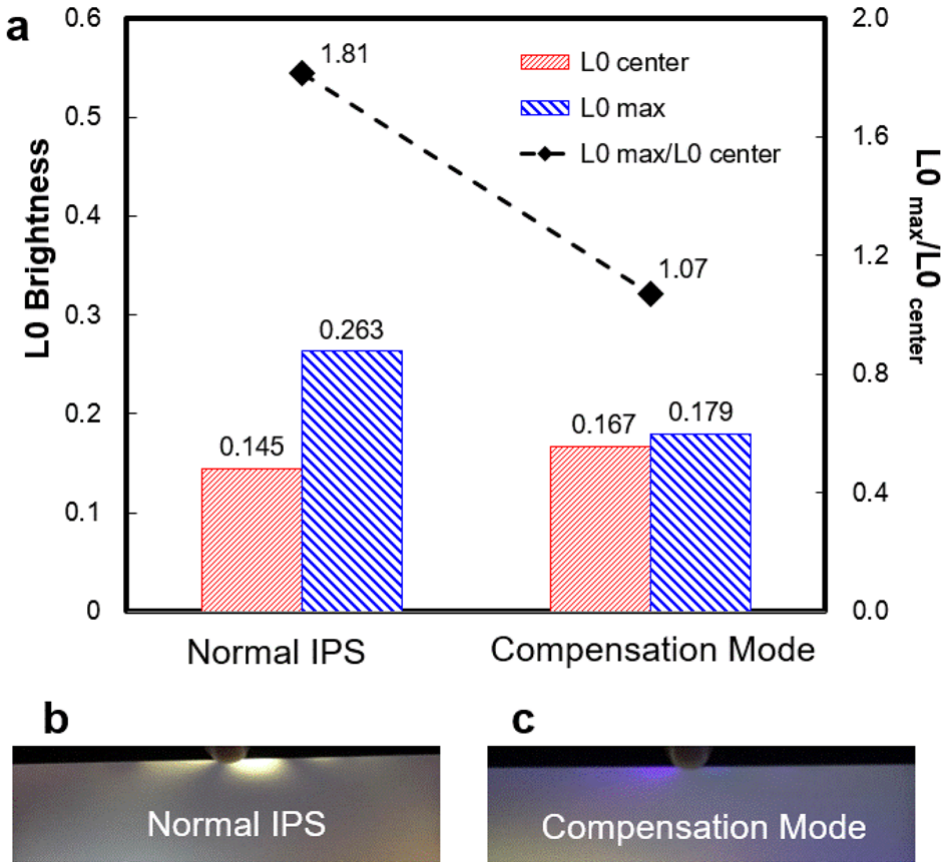
(**a**) The L0 brightness data. (**b**) and (**c**) The photos of LL when the samples are stressed.

Figure 10 is the LL compensation result. It can be seen from Fig. 9a that when the additional stress is received, the brightness ratios of light leakage position (0.265nit) to the center position (0.145nit) is 1.81 for normal IPS, and the brightness ratios of LL position (0.179nit) to the center position (0.167nit) is 1.07 for compensation mode.

As shown in Fig. 10b and c, under the same force, the normal sample has much higher LL brightness than compensation mode. The compensation mode realizes the effect of eliminating LL, but there is still some LL under extra stress. This is because of the dispersion of LC and  +A material. The principle will be explained in detail below.

#### The curved demos

In order to verify the LL elimination effect of compensation technology on curved samples, the samples of 13.3 inches with a curvature of 2800R/2500R/2000R/1500R/1000R are prepared. And the glass thickness of normal IPS and compensation samples are 0.5t/0.5t (TFT/CF glass). The L0 brightness at the center of the panel and at the four corners of the panel are tested respectively. And the LL compensation effect of different curvatures samples are compared and analyzed. The ratio of the brightness of the four corners to the center is used to represent the LL level. The larger the ratio, the greater the brightness of the four corners, and the worse the compensation effect of LL.

**Figure 11:**
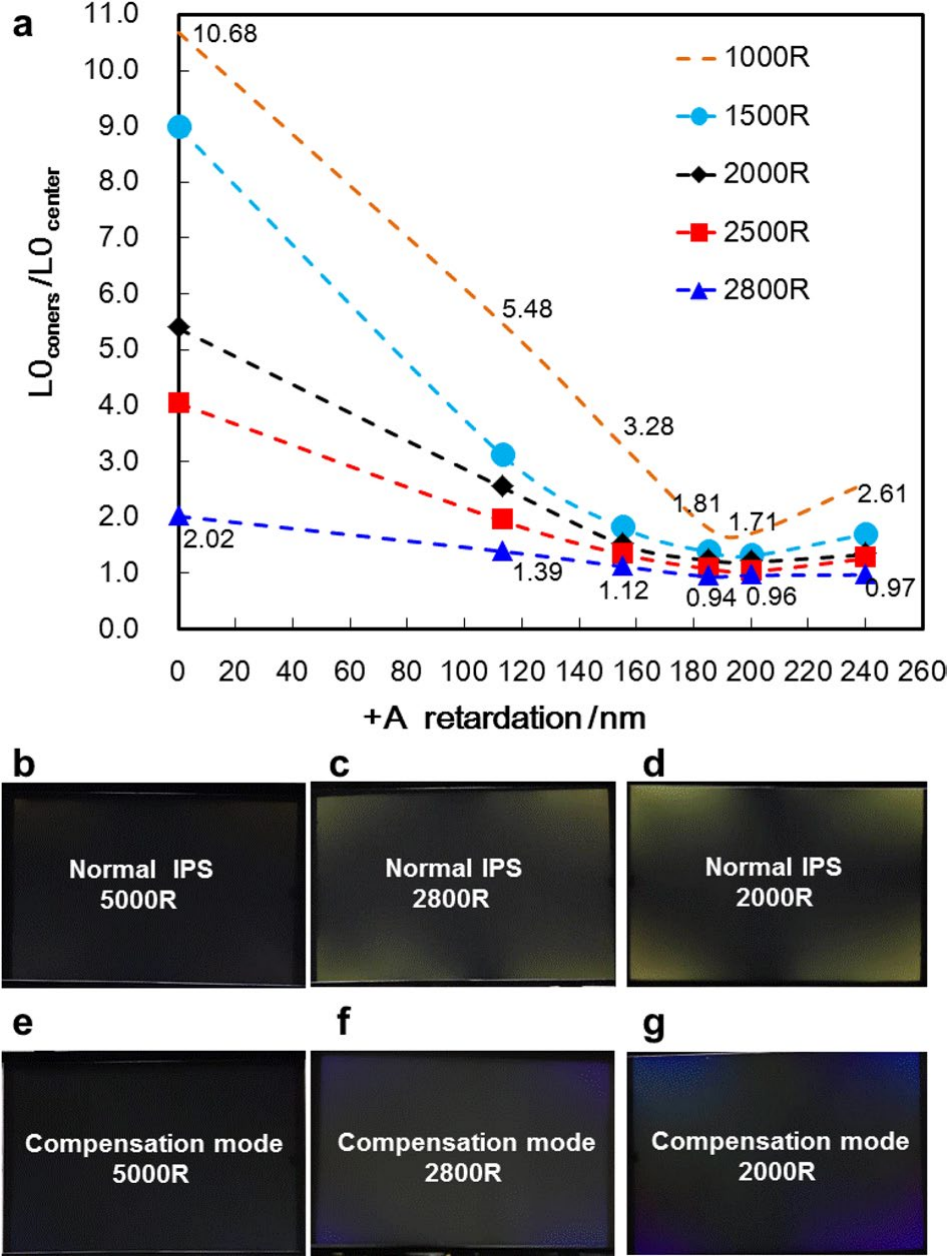
(**a**) The L0 brightness of the curved panels. (**b**–**d**) The photos of curved normal IPS. (**e**–**g**) The photos of curved compensation mode 2.

As shown in Fig. 11a, without compensation, the curved normal IPS has serious light leakage. When the curvatures of demos are 2800R/2500R/2000R/1500R/1000R, the ratios of the four corner brightness to the center brightness are 2.02/4.04/5.38/8.97/10.68, respectively. For the compensation demos, when the retardation of the  +A plate is 200 nm and the curvatures are 2800R/2500R/2000R/1500R/1000R, the LL compensation effects are obvious, the ratios of the four corner brightness to the center brightness are 0.96/1.03/1.20/1.31/1.71, respectively. When the phase retardation of  +A is between 113 and 240 nm, there is a certain effect of LL compensation, and when the retardation value of  +A plate is between 180 and 220 nm, compensation technology can achieve a better LL compensation effect.

In addition, the photos of curved normal IPS and compensation mode 2 at 5000R, 2800R, 2000R are shown in Fig. 11b–g. It can be seen that compensation mode 2 can significantly reduce the brightness of LL compared with normal IPS, it still appears slightly purple and is different from that of the normal IPS. This is also caused by the dispersion characteristics of  +A plate and LC materials, and can be optimized by adjusting the retardation and dispersion characteristics of  +A plate and LC materials.

Consequently, according to the L0 brightness test results of the curved demos above, if the curved demos are prepared with 0.5t/0.5t (TFT/CF) glasses, the four corners of normal IPS demos have serious light leakage, but the compensation mode2 can achieve effective LL elimination. The thickness of 0.5t glass is a commonly used glass thickness in the IPS industry, which can achieve the greatest thickness reduction while meeting the strength of the glass in the manufacturing process. If the glass is thinned, although the stress birefringence of the glass can be reduced, it will also seriously reduce the strength of the glass and Introduce a series of inevitable problems, such as the lower yield rate and the increased cost caused by glass slimming.

## Conclusion

In this paper, a compensation structure with excellent dark state image quality is proposed and experimentally analyzed. This technology can fundamentally improve the dark state LL even under deformation. By introducing a  +A plate that is sandwiched between the glass and the homogeneous LC layer, the LL caused by the combined effect of the phase retardations from the stressed glasses and the LC layer can be eliminated. But the compensation layer for glass stressed LL must be placed between the upper and lower glass, inside the cell. The conventional scheme of using compensation polarizer outside the cell cannot achieve the compensation effect of the scheme proposed in this article. We have proposed two light leakage compensation mechanisms and structures, compensation mode 1 and mode 2. Considering the optical characteristics, especially the effect of CR of  +A on the panel, we recommend the mode 2 solution. For mode 1, after the CR of  +A material is improved, it is also a good light leakage improvement solution. This compensation technology is applicable for IPS modes. In addition to theoretical analysis of compensation principles, we have also developed  +A materials that can meet the preparation process of IPS and prepared effective compensation demos. It is proved that the solution proposed in this paper is not only effective for reducing the local stress LL of flat panels but also effective for weakening the curved stress LL.

## Materials and methods

### Fabrication of  +A plate in compensation

The LC polymer used in this research is the LIXON COAT PLC-75BT series of JNC, involving PLC-75BT01 ~ PLC-75BT07. Among them, PLC-75BT05 is recommended because of its better optical characteristics. At first, the  +A material is coated on CF glass by slit coating, where the viscosity  +A is 3.3 mpa•s and the coating speed is 150 mm/s. Then, put the  +A glass in a vacuum chamber with a pressure of 30pa to remove the solvent. When the set pressure is reached, the glass is placed on the heating plate and kept at 90 °C for 3 min to further remove the solvent and optimize the  +A alignment. After cooling to room temperature, UV cure the  +A glass (500mj/cm2, 365 nm). Finally, the  +A glass was placed on a hot plate at 110 °C for 30 min to fully polymerize. So far, the preparation of the  +A film layer is completed.
